# A disease progression model comparing the long-term mobility and respiratory outcomes of adults with late-onset Pompe disease receiving cipaglucosidase alfa plus miglustat versus alglucosidase alfa

**DOI:** 10.57264/cer-2025-0202

**Published:** 2026-07-15

**Authors:** Amy Dymond, Will Green, Matthew Crabtree, Alasdair MacCulloch, Neil Johnson, Simon Shohet, Vera Gielen, Sophie Clarke, Patrick Deegan, Jordi Diaz-Manera

**Affiliations:** 1 York Health Economics Consortium, York, UK; 2 Amicus Therapeutics UK Ltd, Marlow, UK; 3 Formerly, Amicus Therapeutics UK Ltd, Marlow, UK; Currently, Molecular Economics Ltd, Hitchin, UK; 4 Department of Medicine, Addenbrookes Hospital, University of Cambridge, Cambridge, UK; 5 John Walton Muscular Dystrophy Research Center, Newcastle University & Translational & Clinical Research Institute, Newcastle Upon Tyne, UK

**Keywords:** forced vital capacity, patient-level simulation model, six-minute walk test

## Abstract

**Aim:**

Late-onset Pompe disease (LOPD) is a rare lysosomal disease primarily impacting muscle strength and respiratory function. LOPD has a substantial burden despite the availability of alglucosidase alfa (alg). Patients often require mobility and respiratory support over time. Cipaglucosidase alfa in combination with miglustat (cipa + mig) is one of two more recently approved treatments for adults with LOPD. Given limited data on the lifetime trajectory to mobility and respiratory support in LOPD, a patient-level simulation model was developed to compare the long-term impact of cipa + mig with alg on these outcomes.

**Materials & methods:**

The patient-level simulation predicts lifetime mobility and respiratory disease progression outcomes based on the 6-min walk distance and %predicted forced vital capacity for alg and cipa + mig for the overall LOPD population using available data and assumptions from experienced clinicians. PROPEL/PROPEL open-label extension (NCT03729362) and ATB200-02 (NCT02675465) studies informed outcomes for four years with cipa + mig and one year with alg. French Pompe disease registry data were used thereafter.

**Results:**

Based on the available data and clinical assumptions, the model predicts cipa + mig slows the overall progression of LOPD, allowing patients an additional 2.72 years without mobility or respiratory support compared with alg. People receiving alg may be wheelchair dependent and require invasive respiratory support for an additional 2.57 and 1.55 years, respectively.

**Conclusion:**

Cipa + mig may delay disease progression compared with alg over the lifetime of a patient with LOPD, which would increase the amount of time spent without mobility and respiratory support dependency.

## Plain language summary: A comparison of long-term mobility & respiratory outcomes of two treatments for late-onset Pompe disease

**What is the article about?:**

Late-onset Pompe disease (LOPD) is a rare disease that impacts muscle strength and respiratory function. Enzyme replacement therapies, such as alglucosidase alfa (alg) and cipaglucosidase alfa in combination with miglustat (cipa + mig), can be used to treat LOPD. However, there are limited data on the long-term impacts of these treatments on mobility and respiratory function. This study used a patient-level simulation model to estimate and compare the long-term mobility and respiratory function outcomes for people using these treatments.

**What were the results?:**

The model predicts that cipa + mig slows the overall progression of LOPD when compared with alg. People receiving alg may be wheelchair dependent and require respiratory support earlier than people receiving cipa + mig.

**Why is this important?:**

There are limited studies on the long-term outcomes of people with LOPD. This study estimates that cipa + mig delays disease progression compared with alg over the lifetime of a patient with LOPD. This would increase the amount of time spent without mobility and respiratory support, which may improve quality of life, increase the amount of time patients can spend with friends and family, and improve daily functioning.

Late-onset Pompe Disease (LOPD) is a rare, genetic, autosomal recessive lysosomal disease that is associated with a deficiency of endogenous acid α-glucosidase (GAA) [1]. This enzyme deficiency results in an accumulation of glycogen in tissues, leading to progressive weakness of skeletal and respiratory muscles, which, while variable, can lead to irreversible damage [2]. Initial symptoms include weakness, fatigue and exercise intolerance. Ultimately, an increased dependence on respiratory and mobility support is observed as the disease progresses [3]. This continual decline in mobility and respiratory function is associated with a substantial reduction in health-related quality of life (HRQoL) and increased mortality risk [4,5].

Prevalence estimations of LOPD vary widely and published epidemiological data related to LOPD are limited. The diagnosed birth prevalence is reported to be 1 in 18,711 births worldwide [6,7]. The prevalence estimations of LOPD are within the criteria for a disease to be considered rare in Europe (less than one in 2000 people), and the small population contributes to limited published data informing the lifetime trajectory to mobility and respiratory support in LOPD [8].

The standard of care for LOPD is enzyme replacement therapy (ERT). Alglucosidase alfa (alg) was the first ERT approved for LOPD, and two next-generation ERTs have subsequently been approved (as included in the European Pompe Consortium guidelines) [9]. The use of ERT is also included in the European consensus recommendations [1]. Alg is generally associated with an initial improvement or stabilization in clinical outcomes. However, studies report that patients experience a secondary decline in pulmonary function, motor function and muscle strength after 3–5 years of treatment, and some patients do not respond at all [10,11]. As such, there remains a significant unmet need for an effective treatment particularly for those who meet the following criteria: they do not respond to treatment, the initial response is lost, or they are unable to be treated with alg.

Cipaglucosidase alfa plus miglustat (cipa + mig) is one of the next-generation ERTs, the other being avalglucosidase alfa (aval). Of the two, cipa + mig is unique in being a two-component therapy that combines a cellularly derived bis-M6P-enriched rhGAA enzyme (cipa) with an oral enzyme stabilizer (mig) that is designed to maximize enzyme uptake into skeletal muscle and ensure complete processing into its most active form to cleave glycogen into glucose in the lysosome [5]. Cipa + mig was studied in the Phase III PROPEL trial in adults with LOPD and received regulatory approval in the EU and US in 2023 [12–14].

## PROPEL Trial

The pivotal PROPEL clinical trial (NCT03729362) evaluated the safety and efficacy of cipa + mig compared with alg plus placebo for the treatment of adults with LOPD [14].

Cipa + mig showed an overall improvement in the mean change from baseline in the absolute 6-minute walking distance (6MWD) after 52 weeks in the overall trial population compared with alg. However, the difference did not reach statistical significance. Pre-specified analyses, including those in the ERT-experienced and ERT-naive subgroups, and for other end points, were interpreted as nominal statistical assessments. Cipa + mig showed a nominally significant improvement in sitting %predicted forced vital capacity (%predicted FVC) compared with alg in the overall (combined naive and experienced) and ERT-experienced populations. Cipa + mig also showed a nominally significant improvement in 6MWD compared with alg in the ERT-experienced population. Cipa + mig was generally well tolerated and had a similar overall safety profile to alg. The authors concluded that, based on the totality of evidence from the secondary efficacy end points, cipa + mig showed clinically meaningful improvements in motor function and pulmonary function when compared with alg in the overall and ERT-experienced populations.

Participants in the PROPEL trial could transfer to an open-label extension (OLE) study after the 52-week follow-up [15]. Initial results, at an additional 52 weeks (104 weeks after PROPEL baseline), indicated cipa + mig treatment was well tolerated and associated with maintained improvements in the 6MWD and stabilization in the %predicted FVC [15]. These findings were also observed within the open-label phase I/II study for up to 48 months [16].

## Objective

The objective of the analysis was to model long-term disease progression for people with LOPD, in terms of mobility and respiratory support, when comparing cipa + mig with alg. The analysis was conducted to provide insight into the potential long-term health outcomes because there are limited studies on the lifetime trajectory to mobility and respiratory support in people with LOPD who are treated with alg and cipa + mig [10,11].

## Materials & methods

The current analysis uses a patient-level simulation (PLS) model to estimate the long-term clinical outcomes associated with disease progression among people treated with cipa + mig compared with alg for LOPD.

A cost-effectiveness model was previously developed to estimate the cost-effectiveness of cipa + mig compared with alg for an appraisal by the National Institute for Health and Care Excellence (NICE) in the UK. This modeled long-term health and economic outcomes based on available clinical and real-world evidence. The model underwent rigorous examination and validation throughout the NICE process and was considered to be appropriate for decision-making by the academic External Assessment Group (EAG) and the NICE committee [17]. The disease progression model presented within this manuscript was built to align with the core structure and assumptions of the PLS model submitted to NICE (which were refined further based on clinical input to meet the objectives of the current study). The current model was designed to capture long-term outcomes of people with LOPD globally. Therefore, economic elements were not modeled because they would be specific to the UK.

The modelling approach, inputs and assumptions were validated by two UK-based clinicians who treat people with LOPD across nationally recognized Lysosomal Storage Diseases centers. These clinicians are included in the author list.

### Model structure

**Figure 1.**
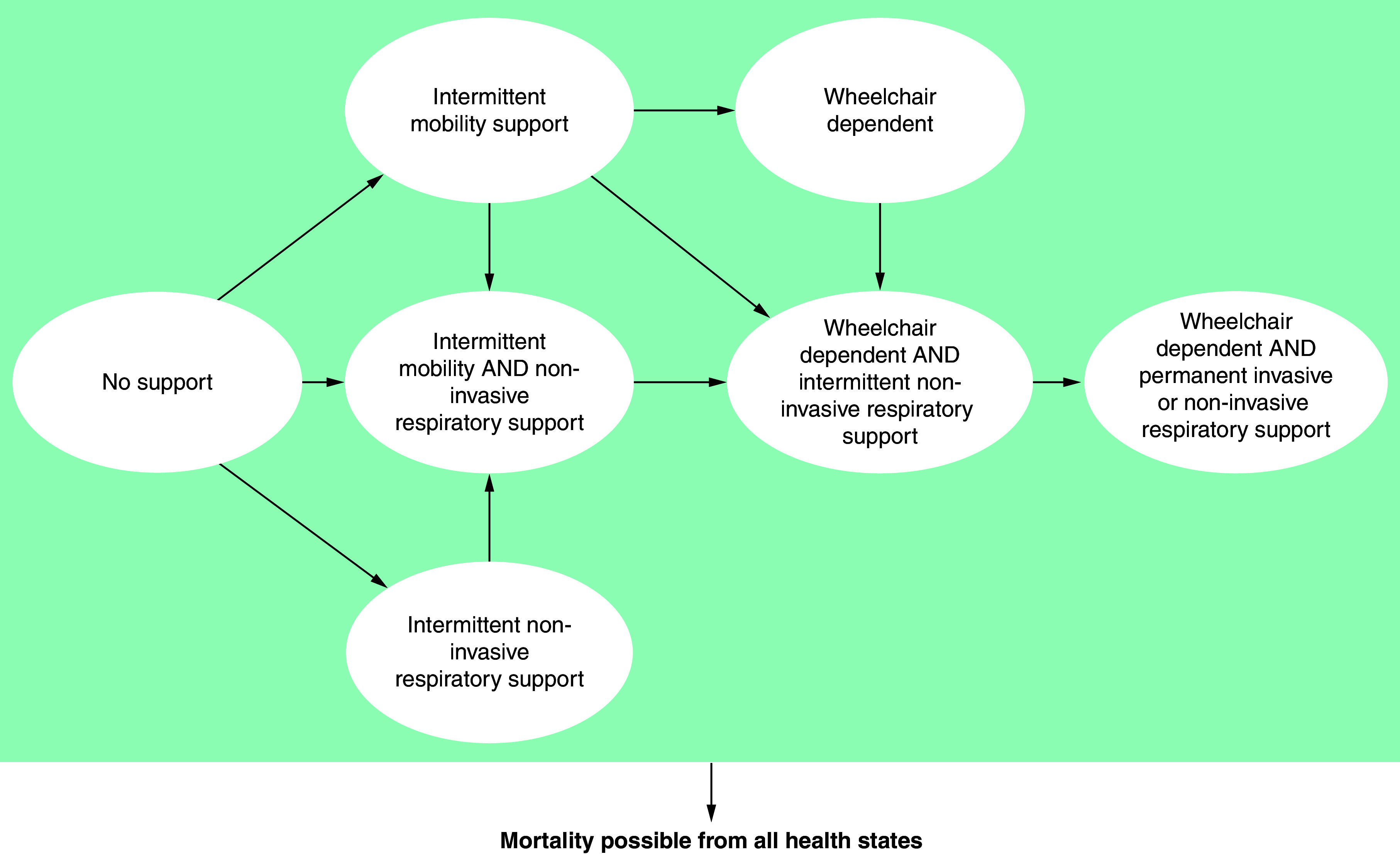
Schematic of the late-onset Pompe disease model structure.

A PLS model (Figure 1) was developed to compare the effect of cipa + mig with that of alg on the disease trajectory of LOPD over a lifetime time horizon. The model was built to align with guidance on patient simulation modeling that was provided in the NICE technical support document on this topic [18].

A hypothetical cohort of people with LOPD entered the model in the first annual cycle [14]. After the first cycle, the model cohort could transition through a small number of health states that were associated with different levels of mobility and/or respiratory support. In terms of mobility support, the following options were captured:

- No mobility support at any time point.
- Intermittent mobility support: Able to walk or move independently with a variety of mobility aids and intermittent use of a wheelchair.
- Wheelchair dependent: Non-ambulant and unable to walk or move independently.

The following respiratory support options were also captured:

- No respiratory support at any time point.
- Intermittent respiratory support: Non-invasive ventilation was required intermittently (usually at night only).
- Dependent on respiratory support: Permanent ventilatory support required.

The health states centered around the mobility and respiratory support requirements because two clinical experts confirmed this was a common measure to gauge the progression of LOPD during individual interviews.

The 6MWD was used to model the decline in mobility, including when patients required intermittent mobility support and became wheelchair dependent. The %predicted FVC was used to model the decline in lung function, including when patients required intermittent noninvasive and permanent invasive respiratory support.

Each person was randomly assigned baseline 6MWD and %predicted FVC value. These values were based on distributions from the PROPEL trial and were considered generalizable to the overall population of people with LOPD [14]. A rate of change in 6MWD and %predicted FVC was assigned to each person within each cycle. The rate of change was treatment-dependent and varied throughout the time horizon (as explained further below). The cycle during which a person's condition had declined to the point of requiring intermittent mobility support/wheelchair dependence and/or respiratory support was determined by specific threshold values for the 6MWD and %predicted FVC, respectively (Table 1 for data inputs).

**Table 1.** Key model inputs – late-onset Pompe disease population data.

	Value	Source	Ref.
**Baseline characteristics**			
Mean age (SD), years	46.80 (13.17)	PROPEL	[14,15]
Mean weight (SD), kilograms	74.71 (18.53)		
Mean (SD) baseline predicted FVC, %	70.5% (0.20)		
Baseline 6MWD (m): estimated from correlation^†^	355.52		
**Thresholds required for mobility and respiratory support**			
Intermittent mobility support, 6MWD (m)	250	UK clinical opinion	
Wheelchair dependent, 6MWD (m)	75		
Respiratory support dependent, predicted FVC, %	30		
Intermittent respiratory support, predicted FVC, %	40	NICE HST23 and TA504	[19,20]
**General population mortality hazard ratios**			
Mean: No mobility of respiratory support	1	Güngör *et al.*	[4]
Mean (95% CI): Mobility support only	2.87 (0.98 to 8.36)		
Mean (95% CI): Intermittent respiratory support only	2.05 (0.62 to 6.77)		
Mean (95% CI): All other health states	5.32 (2.25 to 12.56)		

† Estimated using a regression model with an intercept of 248.18 (if %predicted FVC was 0%) and indicating a statistically significant increase of 1.52m in 6MWD per one unit increase in %predicted FVC.

6MWD: 6-min walk distance; FVC: Forced vital capacity; HST: highly specialized technologies guidance; NICE: National Institute for Health and Care Excellence; SD: Standard deviation.

The model assumed 6MWD and %predicted FVC scores would deteriorate progressively, according to the progressive nature of the disease. People in any health state could also die at any time. A half-cycle correction was used to account for the fact that the transition of people could occur at any point within the annual cycle.

The annual changes in 6MWD and %predicted FVC were simulated independently using a normal distribution for 10,000 people with LOPD over a lifetime time horizon (i.e., from model entry until death). This number was obtained from a stability test undertaken to determine the lowest number of people that could be run through the model to achieve stability, using the methods outlined within the patient simulation modeling decision support unit technical support document [18].

The annual changes in 6MWD and %predicted FVC informed the transition of each person between the health states within the model using annual cycles. The average number of years a person spent in each health state was estimated over a lifetime horizon.

### Model input parameters

Key model input parameters are presented in Table 1, with efficacy parameters presented separately in Table 2. The model inputs were obtained from the aforementioned clinical trial data, where possible, and a targeted literature review was conducted to fill any evidence gaps [14].

**Table 2.** Key model inputs – treatment data.

	Cipa + mig	Source	Alg	Source	Ref.
**6MWD^†^, mean (SE)**					
Baseline to year 1	3.5% (0.008)	PROPEL OLE	1.20% (0.011)	PROPEL	[14,15]
Year 1 to year 2	0.05% (0.01)		1.4% (0.003)	Semplicini *et al.*	
Year 2 to year 3	0% (0.005)	No decline assumed in alignment with outcomes from 4-year Phase I/II study	-2.3% (0.003)		[10,16]
Year 3 to year 4	0% (0.005)				
Year 4 onwards	-2% (0.003)	Relative progression rate compared with alg of 0.85 (assumption supported by UK clinical experts and accepted by NICE)			[17]
**%predicted FVC, mean (SE)**					
Baseline to year 1	-0.93% (0.007)	PROPEL OLE	-3.95% (0.008)	PROPEL	[14,15]
Year 1 to year 2	0.14% (0.008)		-0.9% (0.001)	Semplicini *et al.*	
Year 2 to year 3	0% (0.005)	No decline assumed in alignment with outcomes from 4-year Phase I/II study			[10,16]
Year 3 to year 4	0% (0.005)				
Year 4 onwards	-0.8% (0.001)	Relative progression rate compared with alg of 0.85 (assumption validated by UK clinical experts and accepted by NICE)			[17]

† Absolute 6MWD values were converted to %predicted using the equation detailed in Enright *et al.* (1998) [21] to allow for comparisons between data sources.

6MWD: 6-min walk distance; Alg: Alglucosidase alfa; Cipa + mig: cipaglucosidase alfa plus miglustat; OLE: Open label extension; SE: Standard error.

#### Population

The modeled population aligned with the mean baseline characteristics of the overall population in the PROPEL trial [14]. It was defined as: all adults with a confirmed diagnosis of LOPD (based on documentation of a deficiency of the GAA enzyme or *GAA* genotyping).

The starting age in the model was 46.80 years (SD: 13.27), and 54.5% of the population was female. A summary of further baseline characteristics of the hypothetical simulated model cohort is provided in Table 1.

#### Treatment efficacy data inputs – initial change based on clinical trial data

The probability and/or magnitude of initial improvement and rate of progression differed depending on the treatment received. Data from clinical trials informed the initial change from baseline for up to four years for cipa + mig in the overall population (as presented in Table 2) [14–16]. The initial change from baseline for alg in the overall population was also informed by clinical trial data, but applied for one year only [14].

#### Treatment efficacy data inputs – long-term change with alg

A small number of sources reporting the long-term deterioration of people with LOPD were identified, and these were reviewed to determine the appropriate source to inform the long-term change in %predicted FVC and 6MWD associated with alg in the overall population [10,22–24].

Data from the French Pompe Registry were considered the most robust as this study included the highest number of participants and had the longest follow-up period [10]. The baseline participant characteristics were also considered to be comparable between this study and the PROPEL study. Therefore, the long-term change in %predicted FVC and 6MWD associated with alg in the overall population was informed by a prospective analysis from this registry [10].

While there is heterogeneity across the patient population, current evidence indicates people will experience a deterioration in %predicted FVC over time [10]. The evidence also indicates that people experience an initial improvement in the 6MWD upon commencement of treatment, which is then followed by a decline after one or 2 years [10]. Therefore, an annual decline of 2.3% (SE: 0.003) and 0.9% (0.001) for 6MWD and %predicted FVC, respectively, was applied to people receiving alg from year 2 onwards [10].

An additional scenario analysis using data from the Spanish Pompe registry was undertaken to account for the heterogeneity and uncertainty in the trajectories of long-term outcomes for people with LOPD who have been treated with alg. This study included 113 participants for a mean follow-up of 8.8 years [24]. Data on the estimated yearly decline in absolute 6MWD and %predicted FVC in people who were treated for more than five years were utilized to align with the baseline characteristics of people in the PROPEL study (which had a mean ERT duration of 5.70 years across the overall population). Therefore, this scenario applied an annual reduction of 8.59 m absolute 6MWD and -1.0 %predicted FVC to the alg treatment arm.

#### Treatment efficacy data inputs – long-term change with cipa + mig

There is limited evidence to inform the long-term impact of cipa + mig because it was recently introduced into clinical practice. Therefore, it was assumed that the progression rates of both %predicted FVC and 6MWD would be 15% slower than alg. The EAG and NICE committee considered this assumption appropriate during the aforementioned submission [17]. The clinical experts also noted that this assumption may underestimate the effectiveness of cipa + mig, given the results of the PROPEL study [15]. The rate of progression associated with cipa + mig was a key driver of the model results and was varied within scenario analyses.

#### 6MWD & %predicted FVC thresholds for mobility & respiratory support

Due to a lack of precedence on the most valid values to adopt for the mobility and respiratory support thresholds, Pompe disease clinical experts informed and validated the values for both outcomes. The experts advised that someone with LOPD would generally need to use mobility support and become wheelchair dependent once their 6MWD fell below 250 and 75 m, respectively. These estimates were associated with a degree of variability and uncertainty due to a paucity of published literature. Therefore, scenario analyses were run with alternative threshold values.

The threshold for the 6MWD is dependent on the balance of the participant and, hence, the risk of falls. A higher risk of falls is a precipitant for greater wheelchair use and is correlated with lower 6MWD and/or a reluctance for the participant to complete the test. Therefore, the participant will often stop completing the test before the score declines to zero.

The 6MWD is commonly reported as either an absolute value (m) or %predicted (calculated based on gender, weight, age and height by comparing against population norm values). The efficacy data informing the long-term ambulatory progression of individuals receiving alg were presented as 6MWD %predicted. Therefore, absolute 6MWD outcomes from PROPEL were converted to %predicted values using a validated algorithm to allow the outcome to be consistently analyzed within the model [21]. The coefficients used to inform this reference equation are presented in Table 1.

In the absence of a defined %predicted FVC threshold, it was deemed appropriate to use Duchenne muscular dystrophy (DMD) as a proxy for LOPD. Published literature indicates that people with DMD require respiratory support once their %predicted FVC falls below 30% [19]. However, the aforementioned clinical experts considered this to be too low. For example, evidence indicates that people with idiopathic pulmonary fibrosis require respiratory support once their %predicted FVC falls below 50% [20]. The mid-point of these two values was used (40%) as the threshold for intermittent respiratory support. It was also assumed that people with LOPD would become dependent on ventilation support once their %predicted FVC fell by a further 10%.

#### Mortality

All inputs informing the probability of mortality in each health state were obtained from the targeted literature review. An international observational study conducted to assess the impact of alg on the survival of people with LOPD between 2002 and 2011 was used to inform the risk of mortality associated with LOPD [4]. The risk of death in the ‘no support’ health state was assumed to be equal to the general population mortality rates [25]. The increased risk of death once mobility and/or respiratory support were required, compared with the general population, was captured through the application of a hazard ratio (as presented in Table 1).

### Scenario analyses

A series of deterministic scenario analyses assessed the robustness of the results when different inputs or assumptions were varied.

#### Scenario 1: Cipa + mig long-term progression outcomes 25% slower than alg

An optimistic scenario was included whereby the long-term difference in progression between cipa + mig and alg was closer to the difference observed within the PROPEL study. Data from the PROPEL study were used to inform the efficacy of cipa + mig for four years. The progression rates of %predicted FVC and 6MWD associated with cipa + mig were assumed to be 25% slower than alg after four years.

#### Scenario 2: Cipa + mig no progression for eight years

A second optimistic scenario was included that aligns with observations from the OLE study, whereby the treatment effect of cipa + mig was maintained for eight years [16]. Data from the PROPEL study were used to inform the efficacy of cipa + mig for four years. There was assumed to be no change in %predicted FVC and 6MWD for cipa + mig between years four and eight, and the progression rates of %predicted FVC and 6MWD associated with cipa + mig were assumed to be 15% slower than alg after eight years.

#### Scenario 3: Equal long-term progression outcomes between arms

A conservative scenario was included to recognize the uncertainty associated with the long-term effect of cipa + mig beyond the current clinical trials. Data from the PROPEL study were used to inform the efficacy of cipa + mig for four years. The progression rates of %predicted FVC and 6MWD associated with cipa + mig were assumed to be equal to alg after four years.

#### Scenario 4: Spanish registry data

In this scenario, the progression rates of %predicted FVC and 6MWD associated with cipa + mig and alg after four years were informed from patients receiving alg in the Spanish registry data. The purpose of this scenario was to recognize the heterogeneity between populations and data sources throughout the clinical trials. An annual reduction of 8.59m absolute 6MWD and -1.0 %predicted FVC was applied to the alg arm, and the progression rate of cipa + mig was assumed to be 15% slower than alg.

#### Scenario 5: Lower thresholds required for mobility & respiratory support

A scenario was included whereby the thresholds required for mobility and respiratory support were 20% lower than the values used in the base case. The 6MWD thresholds for intermittent mobility support and wheelchair dependency were reduced to 200 and 60 m, respectively. The %predicted FVC thresholds for intermittent respiratory support and respiratory support dependency were reduced to 32% and 24%, respectively. This scenario was conducted to account for the fact that the transition between health states is multifactorial and not solely informed by the 6MWD and %predicted FVC in clinical practice.

#### Scenario 6: Higher thresholds required for mobility & respiratory support

A scenario was included whereby the thresholds required for mobility and respiratory support were 20% higher than the values used in the base case. The 6MWD thresholds for intermittent mobility support and wheelchair dependency were increased to 300 m and 90 m, respectively. The %predicted FVC thresholds for intermittent respiratory support and respiratory support dependency were increased to 48% and 36%, respectively. As above, this scenario was conducted to account for the fact that the transition between health states is multifactorial and not solely informed by the 6MWD and %predicted FVC in clinical practice.

## Results

### Primary analysis – 15% slower progression of cipa + mig versus alg

**Figure 2.**
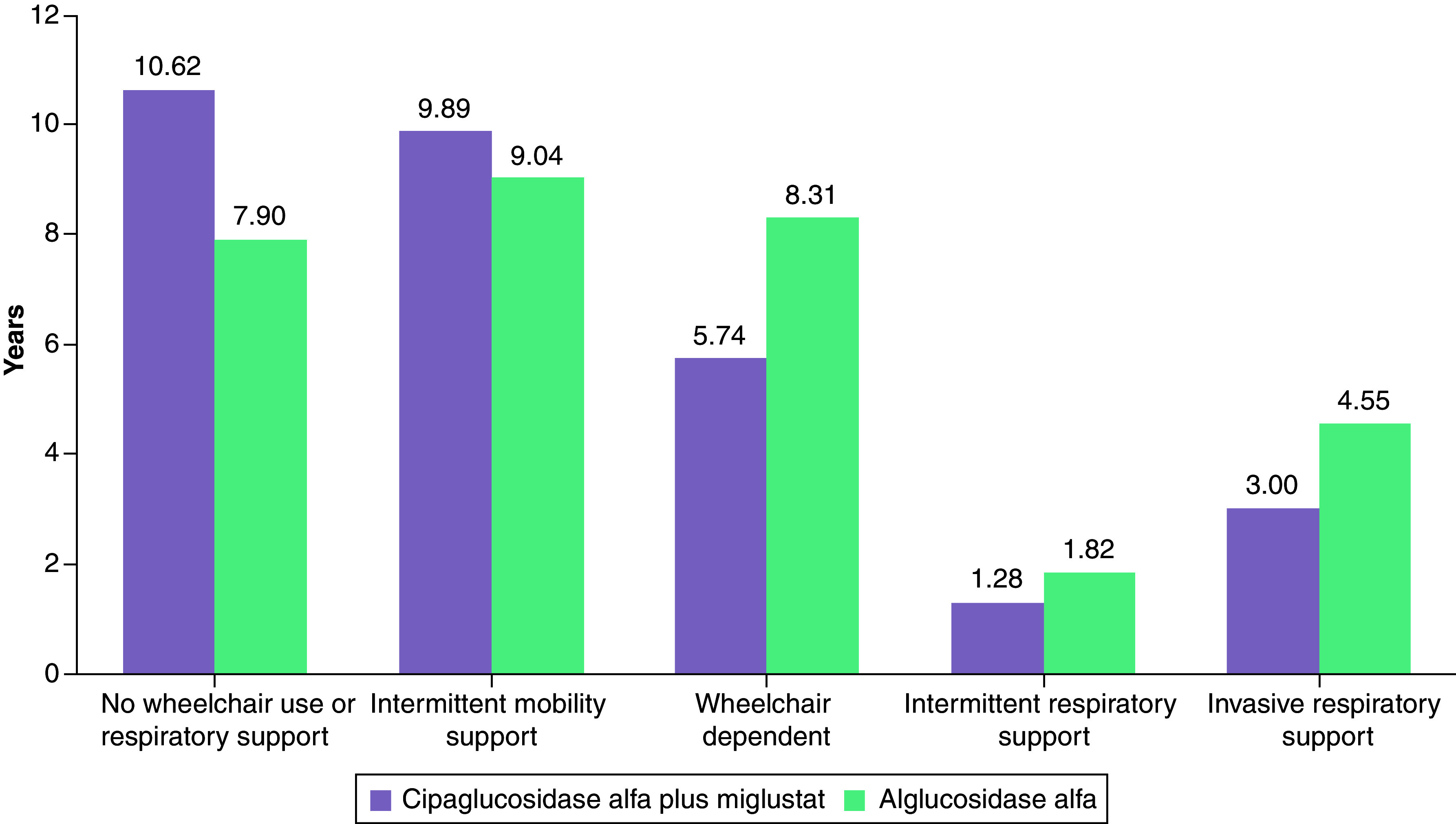
Health state occupancy over a lifetime time horizon in an economic model of late-onset Pompe disease.

The distribution of estimated health state occupancy across the lifetime time horizon is presented in Figure 2. These results are based on the model inputs displayed in Tables 1 & 2. If the model assumptions stand to be true, the model estimates that people receiving cipa + mig spend an additional 2.72 years in the ‘no support’ health state when compared with alg. Instead, people receiving alg may spend an additional 2.57 and 1.55 years in the ‘wheelchair dependent’ and ‘invasive respiratory support’ health states, respectively.

**Figure 3.**
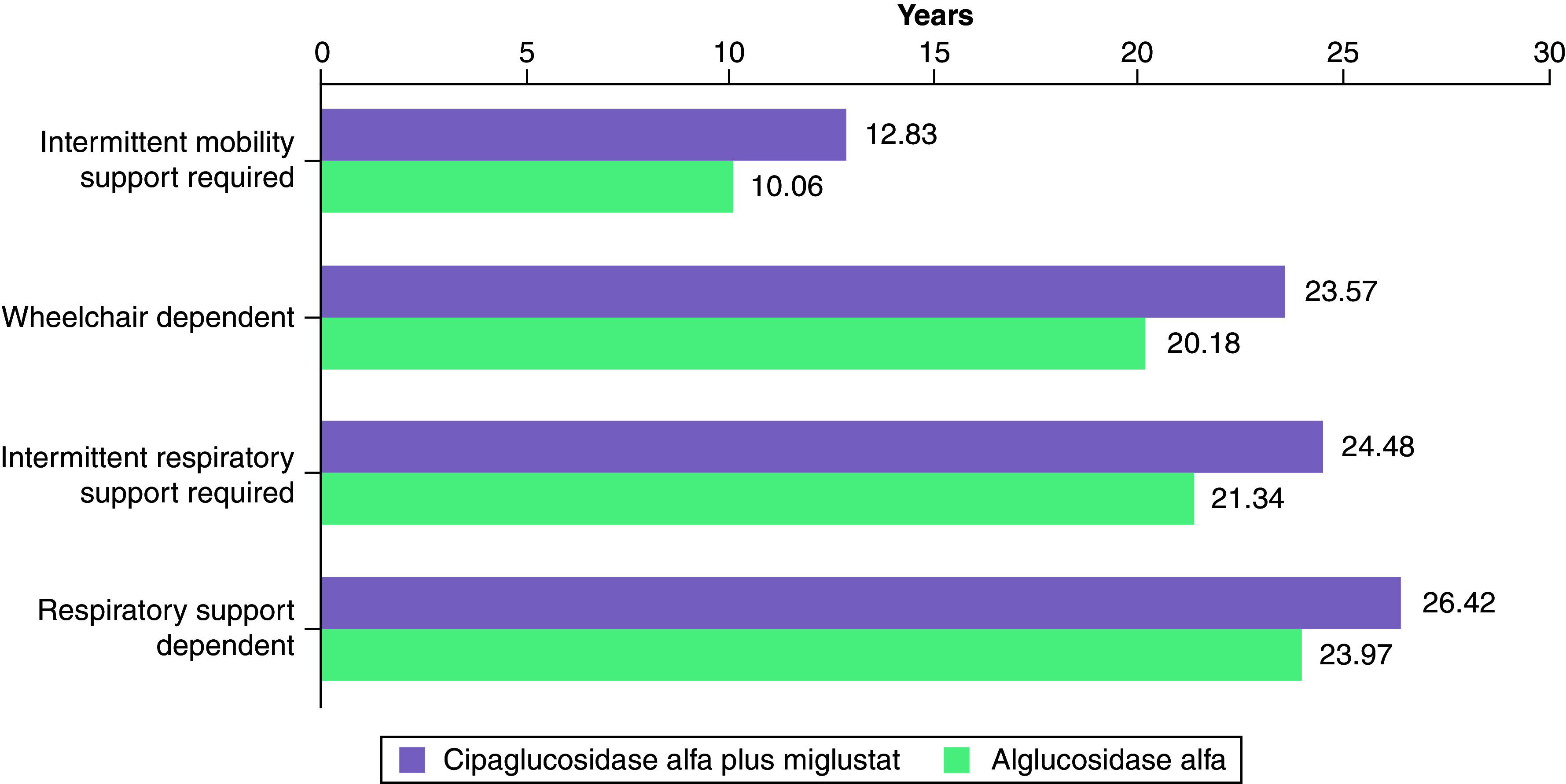
The mean estimated time to mobility or respiratory support since treatment initiation.

The estimated mean time to mobility or respiratory support is presented in Figure 3, which shows that people remain longer without support when receiving treatment with cipa + mig. Based on the model predictions, people receiving cipa + mig require intermittent wheelchair support and become wheelchair dependent 2.77 and 3.39 years later than those receiving alg, respectively. Additionally, people receiving cipa + mig required intermittent respiratory support and became respiratory support dependent 3.14 and 2.45 years later than those receiving alg, respectively.

**Figure 4.**
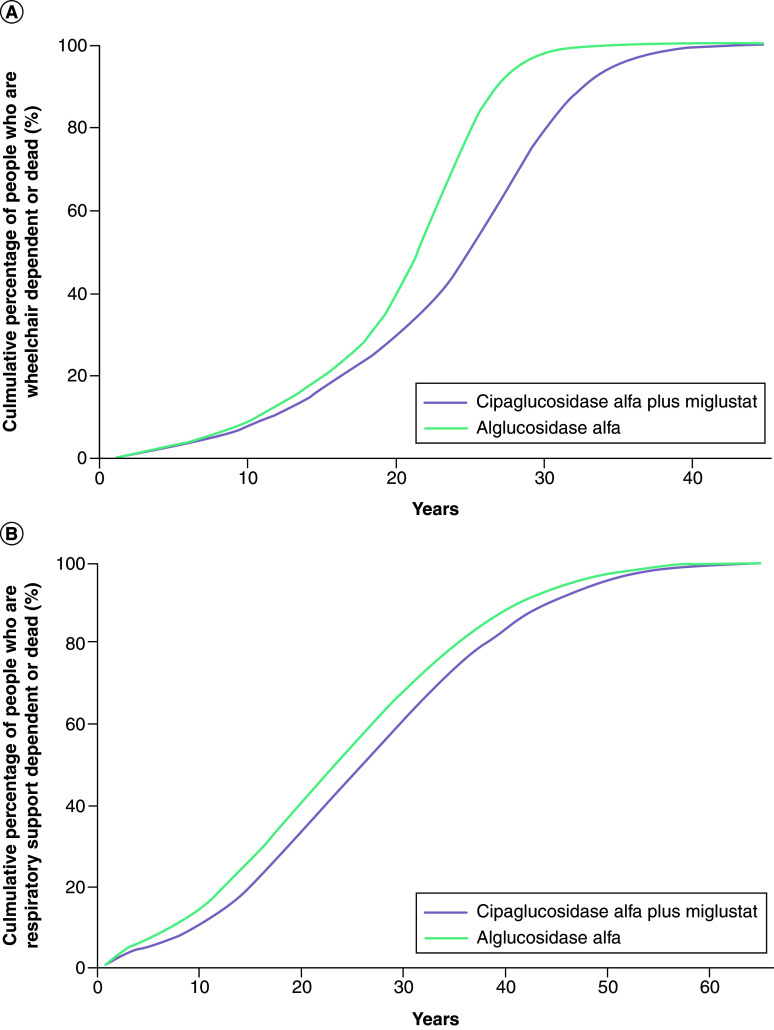
The cumulative proportion of people in different health states (A) wheelchair dependent or dead; **(B)** respiratory-support dependent or dead) since treatment initiation in an economic model of LOPD. LOPD: Late-onset Pompe disease.

The estimated cumulative proportion of people requiring mobility and respiratory support associated with each treatment is presented in Figure 4. The median time to wheelchair dependency is 25 years (25% and 75% percentiles: 18 years and 29 years) and 21 years (25% and 75% percentiles: 16 years and 24 years) for cipa + mig and alg, respectively. The median time to respiratory support dependency is 25 years (25% and 75% percentiles: 16 years and 35 years) and 23 years (25% and 75% percentiles: 14 years and 32 years) for cipa + mig and alg, respectively.

### Additional scenarios

The estimated mean time to mobility or respiratory support time to mobility and respiratory support associated with each scenario is presented in Table 3. People receiving cipa + mig require mobility and respiratory support later than those receiving alg in all scenarios.

**Table 3.** Scenario analyses – average time to mobility or respiratory support (years).

Scenario	Intermittent mobility support			Wheelchair dependent			Intermittent respiratory support			Respiratory support dependent		
	Cipa + mig	Alg	Inc.	Cipa + mig	Alg	Inc.	Cipa + mig	Alg	Inc.	Cipa + mig	Alg	Inc.
Primary	12.83	10.06	2.77	23.57	20.18	3.39	24.48	21.34	3.14	26.42	23.97	2.45
Cipa + mig long-term progression outcomes 25% slower then alg	13.63	10.07	3.56	24.66	20.01	4.65	25.06	21.29	3.77	26.76	23.83	2.92
Cipa + mig no progression for eight years	15.61	10.07	5.53	25.49	20.15	5.34	25.79	21.41	4.37	27.47	24.06	3.41
Equal long-term progression outcomes between arms	11.84	10.02	1.82	21.67	20.11	1.56	23.42	21.28	2.13	25.58	23.93	1.66
Spanish registry data	16.56	12.84	3.72	27.21	24.58	2.63	24.32	20.89	3.42	26.50	23.71	2.79
Lower thresholds required for mobility and respiratory support	16.56	13.32	3.24	24.85	21.34	3.51	27.12	24.37	2.75	28.28	26.09	2.19
Higher thresholds for mobility and respiratory support	8.91	6.85	2.06	22.04	18.87	3.17	21.36	18.08	3.27	24.10	21.45	2.65

Alg: Alglucosidase alfa; CIPA + MIG: Cipaglucosidase alfa plus miglustat; Inc: Incremental difference between cipa + mig and alg.

## Discussion

The current analysis is intended to provide insights into potential long-term health outcomes, in terms of mobility and respiratory status, for people with LOPD treated with alg or cipa + mig. This is relevant because there are limited studies on the lifetime trajectory of people with LOPD across the published literature [10,11].

The model explored the hypothesis that, based on initial short- to medium-term evidence from clinical trials and real-world studies, people receiving cipa + mig are likely to stay stable for longer than those receiving alg and, hence, there will be an increased time until mobility and respiratory support are required.

The outputs from the PLS model support the hypothesis that cipa + mig slows the overall progression of LOPD when compared with alg. People receiving alg required mobility and respiratory support earlier than those receiving cipa + mig in all scenarios, including a scenario whereby there are no progression benefits associated with cipa + mig after four years. The model results also indicate that cipa + mig could delay progression in people with earlier stages of disease to more advanced stages. In particular, the model estimated that people receiving cipa + mig could become wheelchair-dependent and respiratory support-dependent 3.39 (conservative scenario 1.56, optimistic scenario 4.65) and 2.45 (conservative scenario 1.66, optimistic scenario 2.92) years later, respectively, when compared with those receiving alg. For a hypothetical cohort of 100 people, this would mean that cipa + mig generates an additional 339 and 245 years before people with LOPD become wheelchair-dependent and respiratory support-dependent, respectively.

There is a scarcity of data to inform the lifetime trajectory to mobility and respiratory support in people with LOPD who are treated with alg and cipa + mig. Therefore, it was necessary to use assumptions when extrapolating the data and the model was populated using a combination of data from the PROPEL trial, published literature, clinical opinion and assumptions. The results presented throughout this manuscript reflect expected outcomes, if these assumptions stand to be true.

Published literature has shown that important HRQoL losses are associated with reductions in mobility and respiratory function for people with LOPD [5]. Therefore, it is anticipated that cipa + mig also has the potential to contribute to lifetime HRQoL benefits. This was not quantified in the current analysis but could be an area for future research.

To the authors' knowledge, this is the first peer-reviewed publication to estimate the respiratory and mobility support requirements associated with cipa + mig in people receiving treatment for LOPD. However, a previous study compared the difference in the requirements for respiratory and mobility support for people treated with aval compared with alg [26].

The estimations for cipa + mig should be generalizable worldwide because the PROPEL trial recruited participants from 24 countries across the following continents: Europe, North America, Asia and Australia [14]. The PROPEL trial was also reflective of the LOPD real-world population and included a mix of ERT-naive and ERT-experienced patients [10]. Participants within the PROPEL trial were excluded if they required ventilation support for more than six hours per day while awake and were required to have performed two valid 6MWD tests upon randomization [14]. Therefore, the trial captured participants with a moderately severe disease but did not recruit patients at the extremes (i.e., either those who were too mildly affected or those who were too severely affected). A delayed diagnosis may have caused an underrepresentation of patients with mild forms of the disease. However, the baseline characteristics of the PROPEL trial were generalizable to the baseline characteristics of the French registry and the Spanish registry (which was used to inform Scenario 4) [10,24].

The longer-term alg efficacy data, which were used to inform efficacy across all of the cohorts, were derived from a French registry of 197 people – a relatively large study size for a rare disease [10]. It is unknown if any participants were included in both the PROPEL trial and the French registry. There were limited differences between the base case analysis (which was informed by the French registry) and Scenario 4 (which was informed by the Spanish registry). For this reason, it is expected that the long-term outcomes of the PROPEL study would be consistent with these registries.

The key strength of the model is that the structure ensures that the main outcome measures for the LOPD disease studies (i.e., 6MWD and %predicted FVC), which are the key means of measuring progression according to regulators and the clinical community, are fully utilized. The model structure, and corresponding inputs, were reviewed by experienced clinicians and underwent a rigorous assessment process as part of a previous HTA appraisal process [17].

Aval was not included as a comparator in the model. Aval was recently approved in the EU, US and globally [27,28]. The approval varies by country. For example, in the EU and other markets, aval is approved for the treatment of Pompe disease. In the US, and some other markets, the approval of aval is limited to people aged one year and older with LOPD. Next-generation ERTs are gradually replacing alg and this means that a long-term comparison is relevant for decision-making. There are no clinical trials directly comparing cipa + mig with aval and it would not have been possible to include aval as a comparator without adding substantial uncertainty to the model. As aforementioned, a previous study compared the difference in the requirements for respiratory and mobility support for people treated with aval compared with alg [26]. This study found that aval also delays the requirement for noninvasive ventilation, invasive ventilation and wheelchair use when compared with alg (a mean delay of 4.6, 5.6 and 6.4 years, respectively). However, the findings of this study were presented as a poster only, and limited details were available to incorporate aval as a comparator in the model. Furthermore, the COMET study informing this model included a treatment-naive population [29]. The majority of the population in the PROPEL study (∼75%), which informed the present model, were ERT-experienced (mean prior ERT: 7.3 years) [14]. Therefore, the population was not directly comparable to the population within this current model (i.e., an overall population) and the results of the current study are more reflective of a real-world population.

Where possible, robust data sources were adopted to inform model input parameters to ensure appropriate values were applied within the analysis. However, there are many challenges associated with the incorporation of clinical evidence when modeling rare conditions such as LOPD. These include limited trial durations with slowly progressive diseases and a limited understanding of the natural history and epidemiology [30]. The poor availability of long-term data, heterogeneity across the population and a lack of understanding of time to progression are also primary limitations of the clinical data. The impact of these limitations has been explored within the scenario analyses.

Small sample sizes are a primary challenge associated with rare diseases, such as Pompe disease, because few people are eligible to participate [31]. While the PROPEL trial recruited the largest number of participants in an LOPD study to date [14], it should be emphasized that the PROPEL trial did not achieve its primary endpoint for the overall population. Consequently, the incremental benefits of cipa + mig versus alg in the disease progression model were based on numerical or nominally significant differences. However, the evaluation of 6MWD in the ERT-experienced subpopulation (which included the majority of participants) was a prespecified subgroup analysis and was nominally significant. Furthermore, the PROPEL study informed the model results for the first two years of the time horizon only.

Due to the lack of data beyond the completion of the majority of LOPD clinical trials published at the time of the model build, there was uncertainty associated with the long-term efficacy of cipa + mig. The long-term efficacy of this cohort was assumed to be relative to alg due to the absence of alternative information. It was assumed that the long-term progression of cipa + mig was 15% slower than compared with alg, which is in line with the modeling approach adopted in two prior NICE appraisals related to LOPD [17,32].

The PROPEL OLE four-year data showed that patients receiving cipa + mig continued to experience sustained and durable improvements in 6MWD and motor function over four years [33]. Patients also experienced stability or improvement across pulmonary function measures [34]. The long-term progression of alg has been informed from Semplicini *et al.* who reported an annual decline in 6MWD and %predicted FVC of -2.3% and -0.9%, respectively [10]. Therefore, it is a reasonable assumption that, on average, the progression of %predicted FVC would be slower for cipa + mig than alg.

The long-term efficacy assumptions are likely to be conservative in a mixed population given the numerical differences in efficacy between cipa + mig and alg that were observed in the PROPEL trial. It also took longer for people receiving cipa + mig to require mobility and respiratory support than those receiving alg when this assumption was reduced within a scenario analysis. Furthermore, it should be noted that the time to treatment impacts disease progression, and a risk of bias remains because the population was restricted to the characteristics of the PROPEL population, which reflects a moderately severe population.

Due to how the data were presented in the literature (i.e., an average change over several years), a constant subsequent annual change in 6MWD, and %predicted FVC associated with each intervention was applied to each person annually (i.e., a linear change was applied in the longer term during the model time horizon). Given the lack of long-term data, there is a degree of uncertainty associated with this assumption and the trajectory will differ between patients. This uncertainty was captured across the model iterations and the clinical experts all agreed the decline was appropriate and aligned with data observed in the literature [22,24].

All results presented in this manuscript reflect the expected mortality of people with LOPD. The model captured both all-cause mortality and mortality associated with LOPD specifically. It is anticipated that cipa + mig will extend life due to a delay in progression and, hence, reduce complications related to LOPD. However, it was not possible to validate the mortality estimates against published literature due to a paucity of long-term evidence. The relative risk of mortality was also assumed equivalent between the invasive and noninvasive ventilation health states, due to an absence of alternative information. This simplification may underestimate the mortality of people within the invasive health state (especially because respiratory failure is a common cause of death in people with LOPD) and should be updated if data become available in the future.

It was necessary to group the cohort into specific health states to allow the progression of people with LOPD to be modeled; this required simplifying assumptions. In particular, the cohort was stratified depending on the level of mobility and respiratory support required, and specific threshold values for the 6MWD and %predicted FVC were used to determine the point at which a person's condition had declined such that they required support. The level of support required has a continuous nature in reality. However, this was assumed to be dichotomous to enable the transitions across health states to be estimated.

Due to a lack of data within published literature, the values adopted for all health state transition thresholds were not elicited from published literature. Instead, they were informed by information about other diseases (i.e., DMD and idiopathic pulmonary fibrosis) and supported by clinical experts. The clinical experts could not confidently provide a threshold value for the requirement of invasive ventilation. A clinical expert also advised that additional considerations, such as orthopnea, should be used to determine the requirement for ventilation because FVC% does not correlate with the requirement for respiratory support in all cases. However, less common outcomes, such as orthopnea, were not collected in clinical or real-world studies and could not be used to inform the model. Therefore, the results of the analysis are subject to uncertainty.

## Conclusion

In conclusion, based on the model estimations, cipa + mig is predicted to delay disease progression compared with alg over the lifetime of a patient with LOPD, which would increase the amount of time spent without mobility and respiratory support dependency. This may improve HRQoL, increase the amount of time patients can spend with friends and family, and improve daily functioning.

## Summary points

- Late-onset Pompe Disease (LOPD) is associated with symptoms including weakness, fatigue and exercise intolerance; as the disease progresses, this leads to an increased dependence on respiratory and mobility support.
- Alglucosidase alfa (alg) was the first enzyme replacement therapy approved for LOPD and cipaglucosidase alfa plus miglustat (cipa + mig), a next-generation enzyme replacement therapy, has been approved more recently.
- The PROPEL clinical trial evaluated the safety and efficacy of cipa + mig compared with alg plus placebo for the treatment of adults with LOPD.
- The current study used a patient-level simulation model to estimate long-term clinical outcomes associated with disease progression among people treated with cipa + mig versus alg for LOPD.
- The model used the 6-min walking distance to model the decline in mobility and the %predicted forced vital capacity to model the decline in lung function.
- The PROPEL clinical trial and open-label extension informed outcomes for four years with cipa + mig and one year with alg. French Pompe disease registry data were used thereafter.
- The model predicts cipa + mig slows the overall progression of LOPD, allowing patients an additional 2.72 years without mobility or respiratory support compared with alg.
- People receiving alg may be wheelchair dependent and require invasive respiratory support for an additional 2.57 and 1.55 years, respectively.
- By increasing the amount of time spent without mobility and respiratory support dependency, cipa + mig may improve health-related quality of life, increase the amount of time patients can spend with friends and family, and improve daily functioning.
